# The NFI-Regulome Database: A tool for annotation and analysis of control regions of genes regulated by Nuclear Factor I transcription factors

**DOI:** 10.1186/2043-9113-1-4

**Published:** 2011-01-20

**Authors:** Richard M Gronostajski, Joseph Guaneri, Dong Hyun Lee, Steven M Gallo

**Affiliations:** 1 Department of Biochemistry, State University of New York at Buffalo, 140 Farber Hall, Buffalo, NY, 14214, USA; 2 Developmental Genomics Group, New York State Center of Excellence in Bioinformatics and Life Sciences, 701 Ellicott St., Buffalo, NY, 14203, USA; 3 Dept. of Computer Science, State University of New York at Buffalo, Buffalo, NY, 14214, USA; 4 School of Dental Medicine, State University of New York at Buffalo, Buffalo, NY, 14214, USA; 5 Center for Computational Research, New York State Center of Excellence in Bioinformatics and Life Sciences, 701 Ellicott St., Buffalo, NY, 14203, USA

## Abstract

**Background:**

Genome annotation plays an essential role in the interpretation and use of genome sequence information. While great strides have been made in the annotation of coding regions of genes, less success has been achieved in the annotation of the regulatory regions of genes, including promoters, enhancers/silencers, and other regulatory elements. One reason for this disparity in annotated information is that coding regions can be assessed using high-throughput techniques such as EST sequencing, while annotation of regulatory regions often requires a gene-by-gene approach.

**Results:**

The NFI-Regulome database http://nfiregulome.ccr.buffalo.edu was designed to promote easy annotation of the regulatory regions of genes that contain binding sites for the NFI (Nuclear Factor I) family of transcription factors, using data from the published literature. Binding sites are annotated together with the sequence of the gene, obtained from the UCSC Genome site, and the locations of all binding sites for multiple genes can be displayed in a number of formats designed to facilitate inter-gene comparisons. Classes of genes based on expression pattern, disease involvement, or types of binding sites present can be readily compared in order to assess common "architectural" structures in the regulatory regions.

**Conclusions:**

The NFI-Regulome database allows rapid display of the relative locations and number of transcription factor binding sites of individual or defined sets of genes that contain binding sites for NFI transcription factors. This database may in the future be expanded into a distributed database structure including other families of transcription factors. Such databases may be useful for identifying common regulatory structures in genes essential for organ development, tissue-specific gene expression or those genes related to specific diseases.

## Background

Genome annotation, and the ability to extract and use information stored in genome databases, is an essential part of genomic and bioinformatic analysis [1-4]. While now primarily a basic research tool, analysis of genome annotation information is rapidly becoming an important part of Medical and Health Care informatics. As more patient genomes are determined, the ability to correlate changes in the regulatory regions of genes with specific disease states will become increasingly important for Personalized Medicine [5-7].

High-throughput sequencing techniques now allow human and other complex genomes to be sequenced relatively easily [8,9]. However determining the functional significance of sequence changes, particularly changes that affect regulatory elements in the genome, is still in its infancy. The annotation of regulatory elements in genomes has lagged behind the analysis of coding regions [1,2]. While coding regions can be readily assessed by comparing genome sequence with cDNA sequences, regulatory regions are still identified primarily on a gene-by-gene basis. Even with the use of such powerful tools as whole genome ChIP-seq and the Encode project [4,10,11], the functional significance of binding sites found by large scale screening can only be definitively tested by mutational analysis of binding sites within genes and determining the effect of loss of binding on gene expression. Thus, the wealth of published data on the analysis of regulatory elements in genes remains an important asset to be mined by bioinformatic approaches.

Gene expression can be regulated at many levels including control of transcription rate, transcript transport and degradation, translation rate, protein folding and assembly into multi-subunit structures, and protein stability [12]. We have focused on the analysis of cis-regulatory elements as mediators of gene expression [13-17]. In particular, we have created a database for the annotation and analysis of binding sites for site-specific transcription factors in the promoter and enhancer regions of genes. To provide a focus for such a broad topic, we've considered only genes that contain binding sites for the Nuclear Factor I (NFI) family of transcription factors.

The NFI family of transcription factors is essential for the development of multiple organ systems including brain, lung, muscle, hematopoietic cells, and teeth [18-21]. The NFI-Regulome database contains the control regions of genes that have been shown to be regulated by NFI transcription factors in the primary literature. These control regions are annotated with transcription start sites, translation start sites, NFI binding sites, and the location and identity of other known or unknown site-specific transcription factors. Since there are hundreds of known site-specific transcription factors [13,16,17], a comprehensive coverage of all known cis-regulatory sites within a single database is daunting, therefore restricting our analysis to NFI-site containing regulatory regions provides us with a defined starting point for our analysis. Since this gene family has been shown to be essential for a number of developmental processes, the database should also provide useful information on the structures of regulatory regions of genes involved in development and disease.

## Construction and content

### Structure

The database is built using MySQL with the MyISAM engine. This provides the full text support needed for searches. The table structure is designed to be in first normal form, which states that the attributes of the relation contain only atomic values [22]. While third normal form (3NF) can be achieved easily through the use of an algorithm [22], the decomposed tables are not practical for the queries utilized by the website pages. The tables are separated by major characteristics and generally all fields of a given table are utilized by a given query. This enables a single query to retrieve all the information needed for a particular object such as a binding site or a gene. In this case performance concerns outweigh the concerns of anomalies [22] appearing in the relation scheme. Most situations where anomalies can possibly occur are handled through the software due to the limitation of MyISAM not having transaction management or foreign key management.

**Figure 1:**
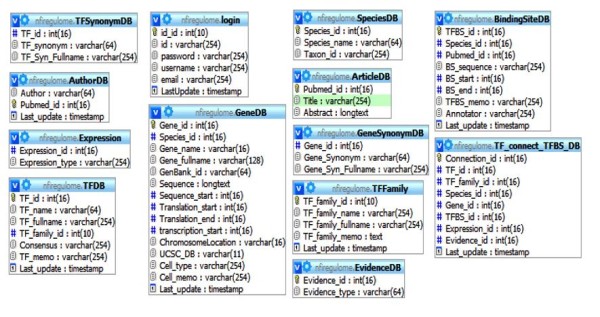
**Table structure of NFI-Regulome Database**. Each table is listed along with the fields and their structures.

**Figure 2:**
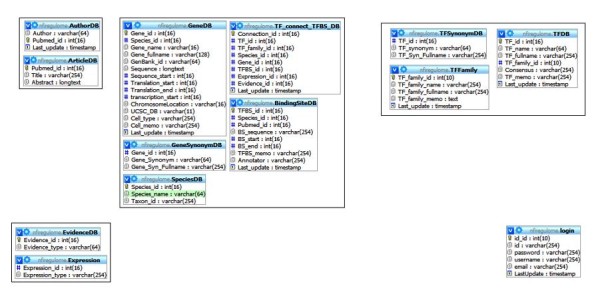
**Structure of NFI-Regulome database grouped by relationships created by the data**. Currently these relationships are controlled by the application layer but in the future will be defined in the underlying database.

The overall structure of the tables (Figure 1) was developed specifically for this database. The tables can be separated into smaller groups which have a complete dependency on each other (Figure 2), two additional tables provide static information, and one table is used for holding user information. Each group is responsible for acting as the data warehouse for a specific set of information.

The AuthorDB and ArticleDB grouping is used for holding information related to PubMed articles. As the PubMed ID is a unique feature, it is used in this grouping as the primary key. The Author field is set currently to only 64 characters maximum as no current value even approaches that maximum. This field maximum can be adjusted if a value were to supersede this arbitrary default. The Abstract field is set to longtext due to the varying length of article abstracts. Utilizing the MyISAM engine allows for searching of this field for keywords at the lowest level which is preferable to creating a piece of software to accomplish the same task.

GeneDB, GeneSynonymDB, SpeciesDB, BindingSiteDB, and TF_connect_TFBS_DB form the main grouping of tables used for holding gene and binding site information. The TF_connect_TFBS_DB acts as a directory to allow a gene to know what binding sites it has and for a binding site to know what gene it belongs to. The GeneDB table includes a Cell_memo field that is used to hold important keywords. The keywords are generally separated by a comma but this is an in-house practice. The use of MyISAM here allows for this field to be searched for string values and can be changed to a text type if the varchar length, currently set to the max of 254, gets exceeded. GeneSynonymDB provides a table that lists the alternative names for a particular gene. While this information could have been listed underneath the Cell_memo field by providing a separate table for this information, fast indexing and access can be provided. This feature can be expanded to other attributes located in the Cell_memo field. The BindingSiteDB table houses all of the binding site information. This table also includes a TFBS_memo field which allows an annotator to list important keywords. In NFI-Regulome these are separated by commas also. Due to the short length of binding site sequences, the binding site sequence field uses a variable type of varchar instead of the longtext used by GeneDB. The BindingSiteDB table also provides the link to the previous group by the inclusion of the Pubmed_id field. The TF_connect_TFBS_DB is the central table of the entire database. The TF_connect_TFBS_DB table connects a particular gene for a particular species to a particular binding site and gives it transcription factor information. Most queries utilized by the NFI-Regulome website reference the information provided in this table as the tables are unaware of how they are related otherwise.

The TFDB, TFSynonymDB and TFFamily tables provide the last major grouping in the NFI-Regulome database. Similarly to GeneDB and GeneSynonymDB, TFDB utilizes TFSynonymDB to house alternative names. TFDB does include a TF_memo name for keywords that provides the same role as Cell_memo and TFBS_memo. TFDB has a TF_family_id field that links TFDB to TFFamily. This relation is also declared by TF_connect_TFBS_DB.

EvidenceDB and Expression contain static information and cannot be changed in software at this time. Values used in these tables have been set and are not expected to change until the next version of the database. Tables were used instead of providing enumerated fields for this information as changes can be made more easily if they need to be changed in the future.

### Functions of the Database

The NFI-Regulome database was designed to fulfill multiple functions: 1) to act as a clearing house and storage database for all genes known from the primary literature to be regulated by NFI transcription factors, 2) to allow rapid analysis and display of defined groups or sets of NFI-regulated genes, 3) to enable rapid comparisons of the size, composition, and organizational structure of the cis-regulatory regions of NFI-regulated genes, selected either by disease-relevance, cell, tissue or developmental stage where the gene is expressed, or on the presence of other transcription factor binding sites, 4) to provide output to other TF binding site annotation databases such as OregAnno, and 5) to be a prototype database for a comprehensive all-transcription factor Regulome database (see discussion). Each of these functions, along with how they are performed, is discussed below.

### Populating the NFI-Regulome database

Literature references on NFI-regulated genes can be input automatically from Pubmed with a Perl script, or can be added individually. Information on the cis-regulatory regions of genes of NFI-regulated genes is input by trained Gene Annotators. The papers are read and a listing of all TF binding sites, transcription start sites and other relevant information including the binding site locations and sequence, tissue or cell-type where the gene is expressed and disease relevance is recorded. The gene sequence is obtained using the UCSC Genome Browser and is input into the database. Due to ambiguous or multiple transcript start sites, the translation start site is used as a defined anchor. A semi-automated sequence editor and search function is provided to locate the specific binding sites for each TF in the regulatory region of each gene. As of 5/20/2010 there are 70 partially or fully annotated genes with 390 annotated sites and 574 NFI-related references in the database. Sites are identified as either experimentally confirmed, or predicted. The vast majority of sites in the database are experimentally confirmed. The sizes and locations of annotated regions correspond to those identified in the specific literature references and include both promoters, enhancers, and silencer regions. All sites are currently from individual research papers and no data from large-scale ChIP have been used. No such large scale studies have been performed to date for NFI transcription factors. Such data will be used when available.

## Utility and Discussion

### Searching the database and displaying information: Basic Search Page

**Figure 3:**
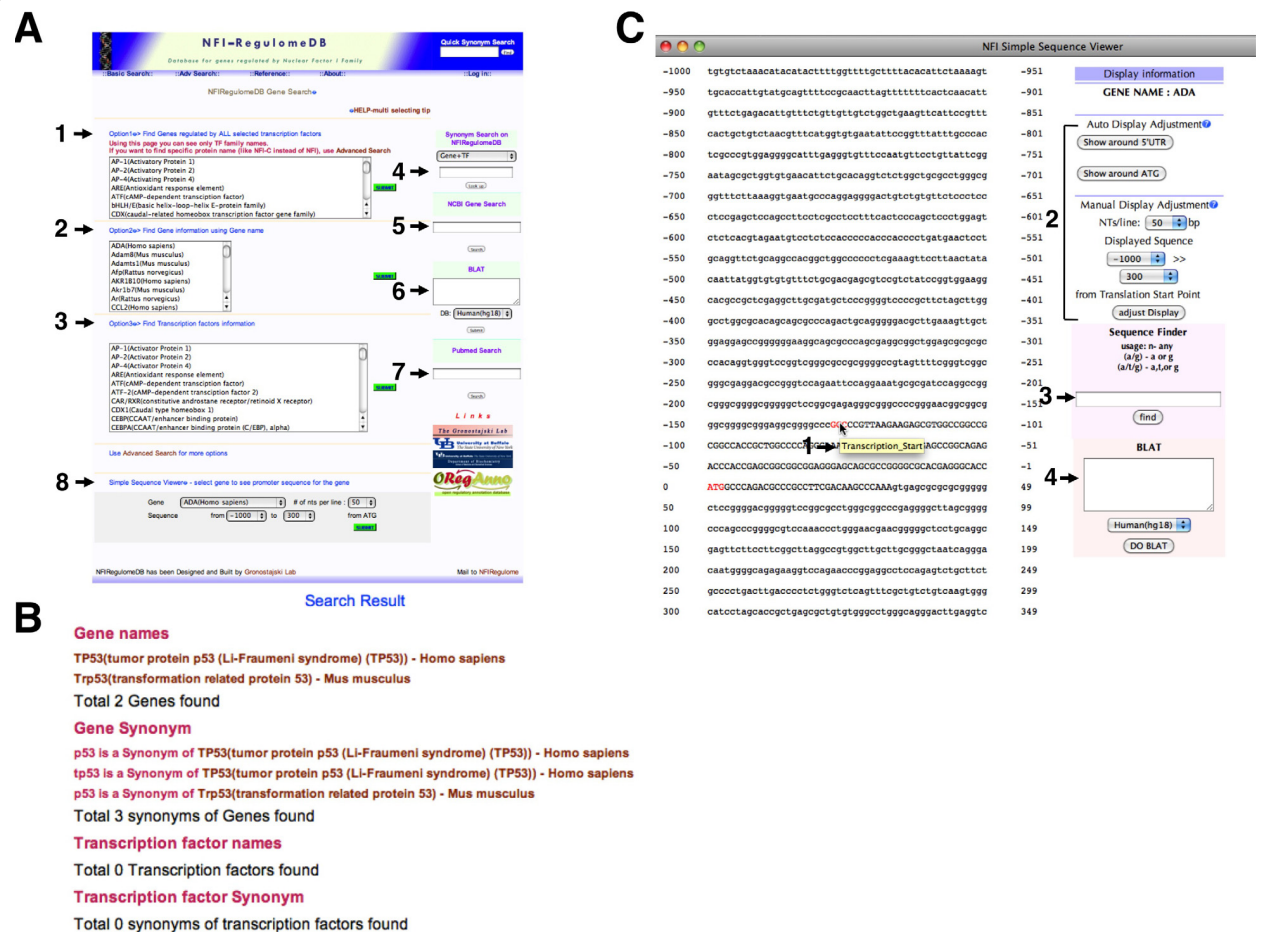
**Basic Search Page of NFI-Regulome Database**. A) Basic Search Page showing options for searching. B) Result of search for P53 in Synonym Search. C) Example of Simple Sequence view.

The home page of the database is also the Basic Search page (Figure 3A). It is anticipated that two major search types will be performed: 1) searching for specific TF binding sites and outputting all genes containing these sites and 2) searching for genes expressed in specific organs, tissues, cell types or diseases. These are accomplished through the Basic Search (Figure 3A) and Advanced Search windows (below), respectively. In the Basic Search window the user has several options: 1) choose a particular TF family or multiple families and display all genes containing binding sites for those families (Figure 3A, arrow 1, Option 1), 2) chose a specific gene or genes and display all binding sites on those genes (Figure 3A, arrow 2, Option 2), 3) choose a specific TF listed and show all genes containing sites for that TF (Figure 3A, arrow 3, Option 3). On the right side of the basic search page one can search for TFs and genes in the database based on commonly used synonyms if the standard gene or TF names are not known (Figure 3A, arrow 4, Synonym Search). For example inputting P53 opens a window showing all P53 genes in the database (Figure 3B). Note that while p53 is indeed a TF, none of the genes in the database contain known binding sites for p53 and therefore it is listed here only as a gene. Clicking on the gene link in the search menu will display binding site information for the gene.

From this page one can also perform searches of NCBI for gene names (Figure 3A, arrow 5, NCBI Gene Search), perform a BLAT search for a specific sequence at the UCSC Genome Bioinformatics site from selected genome database builds (Figure 3A, arrow 6, BLAT), or perform a free text search of Pubmed to find articles related to specific genes or TFs (Figure 3A, arrow 7, Pubmed Search).

The Simple Sequence Viewer is used to display a selected sequence region of a single gene (Figure 3A, arrow 8, Simple Sequence Viewer) with binding sites shown in red (Figure 3C). Placing the cursor over a site in the window will display information on the site (Figure 3C, arrow 1, transcription_start). In addition from the viewer one can change the regions displayed (Figure 3C, bracket 2) and search for specific short sequences within the displayed sequence (Figure 3C, arrow 3, Sequence Finder). From this page the user can also perform BLAT searches of sequences input by either typing or cutting and pasting (Figure 3C, arrow 4, BLAT).

### Binding site displays

**Figure 4:**
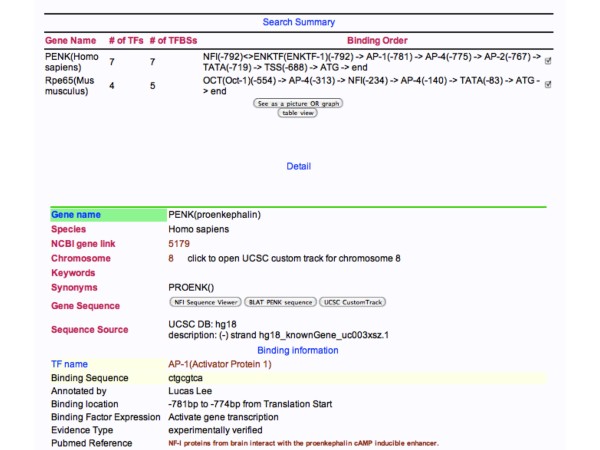
**Search Summary of a search for genes containing sites for the AP-4 family of TFs**. The genes are presented at the top of the page with the number of TFs and TF binding sites (TFBS) and a list of the sites and their locations. In detail, every site is listed along with its location, sequence, whether the site activates or represses expression and whether the site has been experimentally verified. The display is truncated at the bottom of the 1^st ^binding site of PENK.

**Figure 5:**
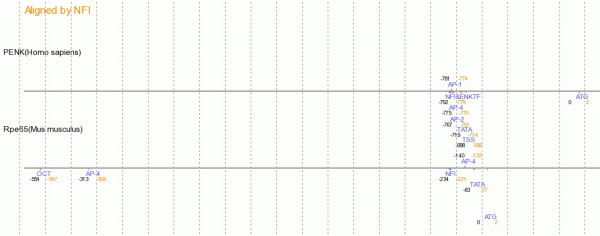
**Picture view of genes aligned by NFI site**. The regulatory regions are stacked horizontally with the binding sites listed above or below each region. Colored wedges denote the relative orientation of each site when known.

**Figure 6:**
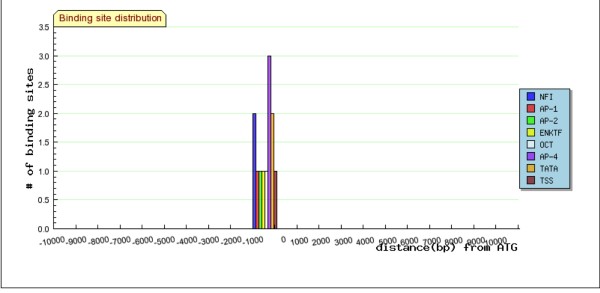
**Graph View of genes containing AP-4 binding sites, aligned by ATG as 0**. The height of the bar indicates the number of binding sites of each type within the bin of 1000 bp. Bins can be adjusted in size.

**Figure 7:**
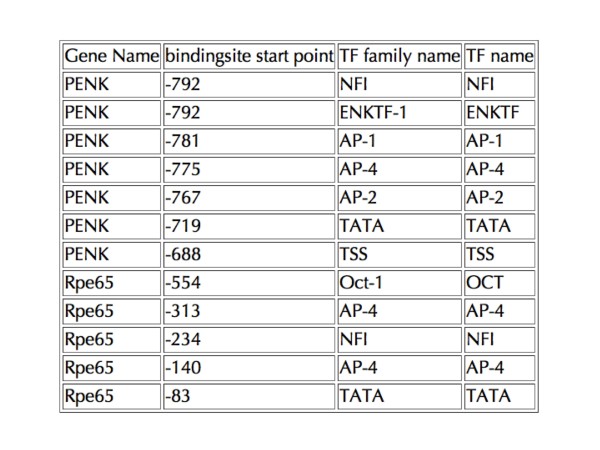
**Table view of genes returned by query for genes containing AP-4 binding sites**. The Gene Name, location of the binding site, TF name and TF Family name are generated and displayed by the database. This table can be used to create modified displays of the sites or for the calculation of relative locations and distributions of binding sites using other software.

Binding sites for TFs can be displayed in a number of ways. Selecting and submitting a TF family or gene returns a display of the location of binding sites on the single or multiple genes with a detailed listing of each binding site shown below the summary (Figure 4). To obtain a visual comparison of the genes either the picture OR graph view, or the table view can be used. The picture view generates an image of each regulatory region displayed one below the other, aligned by one of the transcription factors selected (Figure 5). The Graph view gives a graphical distribution of all the binding sites relative to one selected site (Figure 6). This allows one to visualize the relative distributions of all of the binding sites on the set of genes selected. The table view returns a simple table of all sites and their locations relative to the ATG of the gene (Figure 7). Thus, these different views allow one to either compare each regulatory region with the others, or produce a combined distribution of all binding sites on all the genes within the set.

### Advanced Search page

**Figure 8:**
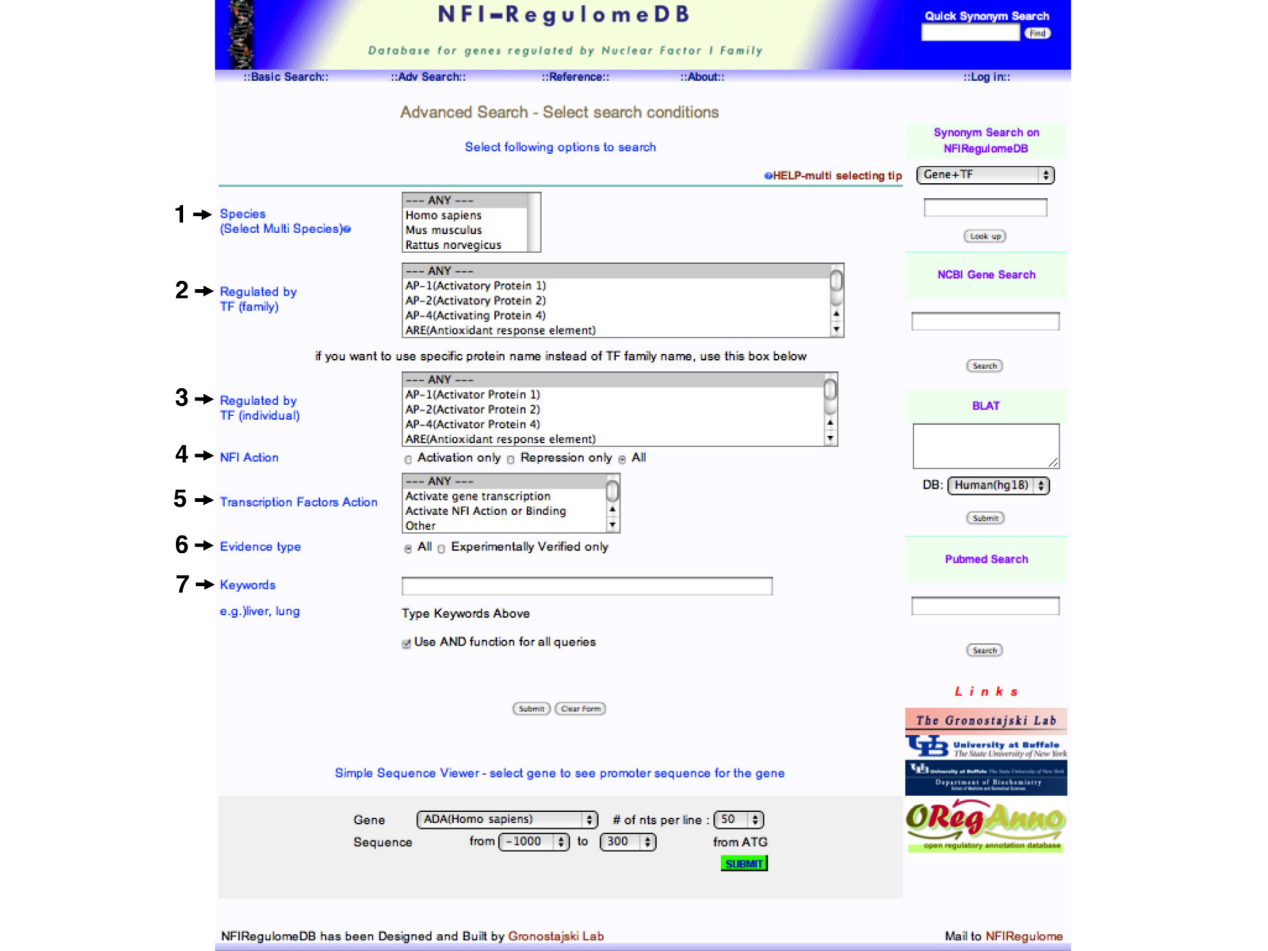
**Advanced Search Page**. This page allows the selection of genes based on multiple criteria including species, TF, whether a gene appears activated or repressed by NFI or other TFs, the evidence for binding and keywords. The keyword search can identify the organ or cell type of expression and disease-relatedness of a gene if these data are input by annotators.

Here one can search for sets of genes by: 1) species (Figure 8, arrow 1, Species), 2) those containing sites for a specific TF family (Figure 8, arrow 2, Regulated by TF (family), 3) those containing sites for a specific member of a TF family (Figure 8, arrow 3, Regulated by TF (individual)), 4) those either activated or repressed by NFI or other TFs (Figure 8, arrow 4, NFI action and arrow 5, Transcription Factor action, respectively), 5) the type of Evidence for binding (Figure 8, arrow 6, Evidence type) or 6) those genes expressed in specific cell types, tissues or disease states (Figure 8, arrow 7, keywords). Currently the keyword search is used to classify many characteristics of the genes, but this is likely to change in future versions of the database. This page contains a link to the same Simple Sequence Viewer and also allows the same searches of TF and Gene synonyms, NCBI, UCSC and Pubmed as those on the Basic Search Page.

### Interactions with other Bioinformatics sites

In addition to the NCBI, UCSC and Pubmed searchs shown above, the user can generate an XML file suitable for incorporation into OregAnno. This feature has been used to distribute binding site information to OregAnno. Users can also generate gff files that allow the sites for selected genes to be displayed on the UCSC Genome browser at the UCSC Bioinformatics site.

### Proposed uses of the database

In its current form the database can be used to answer such example queries as: 1) what are all the genes expressed in liver that contain both NFI sites and CEBP sites, or 2) what are all the genes that contain both GR, AP-1 and NFI binding sites? Regulatory regions containing these specific sites can then be easily compared in the picture view or table view to assess the relative distribution and spatial configuration/orientation of the sites within the regulatory elements. When populated with larger numbers of genes, the statistical significance of the distributions seen could be obtained. In addition, genes associated with specific disease states can be obtained and their regulatory regions compared. As the regulatory regions of more NFI-regulated genes are examined, common features of regulatory regions that contain NFI sites may well be discovered. In addition, specific classes of TFs associated with the expression of genes in specific tissues, cell types, stages in development or disease states can be determined.

### Future growth and management of the database

The current structure of the database is well-suited to the task at hand. Moving forward, the schema of the database will evolve in order to provide new features. These include a method for allowing members of the community to enter gene and regulatory region information, the addition of a Disease-relatedness table, and the refactoring of the database to improve the maintenance of relationships between regulatory regions and annotation information.

Providing a method for members of the community to curate data shown in the literature is important as it allows multiple users from within the community to enter information into the NFI-Regulome database without requiring those users to be located in close proximity to the maintainers. The system will allow members of the community to be provided with curator accounts, thereby allowing them to enter annotation information into the database. Entries provided by community curators will be placed in an "approval queue" where the administrator will provide oversight of the curated information and will have the ability to make any necessary changes/edits to the curated information before it is approved and incorporated into the dataset.

The underlying database will be refactored to utilize the referential integrity constraints provided by the underlying relational database. Referential integrity will add enforceable constraints between related entries in the database and will ensure that the state of the data remains consistent [23]. This functionality is currently provided by the software layer of the NFI-Regulome database and by taking advantage of the features provided by the underlying database we define these constraints at the same time that the data and relationships are defined, freeing the application developer from the need to enforce the constraints and reducing the probability of errors and/or inconsistent information in the data.

### Comparison to other TF binding site databases

The goals and features of the NFI-Regulome database appear unique among TF binding site databases. There are a number of databases that are significantly larger than the NFI-Regulome Database as assessed by the number of binding sites annotated including TRANSFAC [16], JASPAR [17], ORegAnno [3] and the ENCODE contribution to the UCSC Genome Browser [4]. Species- and Kingdom- specific TF binding site databases include RedFly (*Drosophila*) [24,25], RegPrecise (prokaryotic) [26], PlantPAN [27] and GRASSIUS [28], but none of these contain mammalian TFs. The TIGER database [29] is perhaps most similar to the NFI-Regulome database in that tissue-specificity of gene expression is searchable and lists of TF binding sites are shown. Also, TIGER generates lists of co-occurrence of TF binding sites that may be biologically relevant. However the binding sites in TIGER are predicted sites and their functions have not been experimentally verified. In addition, none of these databases can be conveniently queried for sets or combinations of TFs on individual genes, display of their precise locations within the genes, or disease relatedness of a given gene. Thus in these types of queries, and in the ability to display multiple genes aligned by specific TF binding sites, the NFI-Regulome Database provides a unique resource. We are currently working to both increase the number of genes annotated in the database and to improve the annotation features and abilities of the database.

The rate limiting step for input of data into the database is the manual reading of papers by annotators and their reformatting of the published sequence positions for sites to the UCSC coordinates. Because these steps are labor-intensive, we have restricted our current database to the NFI transcription factors. However, we have produced a "generic" database module that other laboratories can use to annotate sites for transcription factors of interest and it is available upon request. We hope eventually to produce a distributed database "cloud" whereby other transcription factor families can be queried for their cognate genes, site location, tissue of expression and promoter/enhancer architecture from a single website.

## Competing interests

The authors declare that they have no competing interests.

## Authors' contributions

RMG developed the concept of the database, determined many of the fields to be included, supervised both annotators and the work of other authors and wrote much of the manuscript. JG contributed to database design and function, performed maintenance and updating of database functions and contributed to writing the manuscript. DHL wrote the PHP and perl scripts to annotate and populate the database and worked on database and table design and interaction. SMG contributed to manuscript preparation and future database design. All authors have read and approved submission of this work.
